# Functional Voltage-Gated Sodium Channels Are Present in the Human B Cell Membrane

**DOI:** 10.3390/cells11071225

**Published:** 2022-04-05

**Authors:** Adam Feher, Marianna Pócsi, Ferenc Papp, Tibor G. Szanto, Agota Csoti, Zsolt Fejes, Béla Nagy, Balázs Nemes, Zoltan Varga

**Affiliations:** 1 Department of Biophysics and Cell Biology, Faculty of Medicine, University of Debrecen, H-4032 Debrecen, Hungary; feher.adam@med.unideb.hu (A.F.); papp.ferenc@med.unideb.hu (F.P.); szantogt80@gmail.com (T.G.S.); jelena45@gmail.com (A.C.); 2 Department of Laboratory Medicine, Faculty of Medicine, University of Debrecen, H-4032 Debrecen, Hungary; pmarcsi89@gmail.com (M.P.); fejes.zsolt@med.unideb.hu (Z.F.); nagy.bela@med.unideb.hu (B.N.J.); 3 Department of Organ Transplantation, Faculty of Medicine, Institute of Surgery, University of Debrecen, H-4032 Debrecen, Hungary; nemes.balazs@med.unideb.hu

**Keywords:** B cell, lymphocytes, voltage-gated sodium channel, patch-clamp electrophysiology, tetrodotoxin

## Abstract

B cells express various ion channels, but the presence of voltage-gated sodium (Na_V_) channels has not been confirmed in the plasma membrane yet. In this study, we have identified several Na_V_ channels, which are expressed in the human B cell membrane, by electrophysiological and molecular biology methods. The sensitivity of the detected sodium current to tetrodotoxin was between the values published for TTX-sensitive and TTX-insensitive channels, which suggests the co-existence of multiple Na_V_1 subtypes in the B cell membrane. This was confirmed by RT-qPCR results, which showed high expression of TTX-sensitive channels along with the lower expression of TTX-insensitive Na_V_1 channels. The biophysical characteristics of the currents also supported the expression of multiple Na_V_ channels. In addition, we investigated the potential functional role of Na_V_ channels by membrane potential measurements. Removal of Na^+^ from the extracellular solution caused a reversible hyperpolarization, supporting the role of Na_V_ channels in shaping and maintaining the resting membrane potential. As this study was mainly limited to electrophysiological properties, we cannot exclude the possible non-canonical functions of these channels. This work concludes that the presence of voltage-gated sodium channels in the plasma membrane of human B cells should be recognized and accounted for in the future.

## 1. Introduction

Voltage-gated Na+ (Na_V_) channels are best known for initiating action potentials by depolarizing the membrane of excitable cells. Na_V_1.1–1.9 are the nine members of this family [1,2], of which Na_V_1.1–1.3 and 1.6 are mostly expressed in the neurons of the central nervous system but can also be found in peripheral neurons, while Na_V_1.7–1.9 channels are almost exclusively found in the peripheral neurons. Na_V_1.4 is the main Na_V_ channel of skeletal muscles, while Na_V_1.5 is expressed in cardiomyocytes [3]. A well-known blocker of Na_V_ channels is tetrodotoxin (TTX), isolated from pufferfish [4,5]. Na_V_ channels are classified as TTX-sensitive (Na_V_1.1–1.4 and 1.6–1.7), with an IC_50_ in the low nM range, or TTX-resistant (Na_V_1.5, 1.8 and 1.9), being blocked only in the µM concentration range [6].

Despite their main function in action potential generation, Na_V_ channels have been found in numerous non-excitable cell types, including astrocytes [7,8,9,10], islet β-cells [11], keratinocytes [12], endothelial cells [13], dendritic cells [14], macrophages and microglia [15,16,17], red blood cells [18], T cells [19,20] and neutrophils [21]. The functions associated with them were often confirmed by TTX application. These functions also span a wide range including membrane potential control, signaling, cytokine secretion, migration, phagocytosis, adhesion and chemotaxis [10,13,14,15,16,17,21]. Moreover, Na_V_ channels are significantly upregulated in many different cancer types, and their function has been linked to invasion and metastasis [22]. 

The expression level of Na_V_ channels can dynamically change in non-excitable cells during differentiation, as was described for the maturation of oligodendrocyte precursor cells [23] and by our own laboratory for the differentiation of dendritic and chondrogenic cells [24,25].

B lymphocytes are one of the main pillars of the adaptive immune system and are responsible for humoral antibody responses. In addition, they also take part in cytokine secretion, the modulation of T cell differentiation and antigen-presentation [26,27,28]. Most studies exploring the channelome of lymphocytes focused on T cell ion channels or analyzed mixed lymphocyte populations, in which B cells are underrepresented, so currently the ion channel repertoire of B cells is less well-defined.

Lymphocytes express a variety of ion channels, responsible for cell development, proliferation, signaling and cell activation. These include voltage-gated, Ca^2+^-activated and background potassium channels, voltage-gated and store-operated calcium channels, a voltage-gated proton channel and transient receptor potential channels [29,30,31,32]. The best characterized K_V_1.3 potassium channel, found both in T and B cells, is activated by membrane depolarization, and its canonical function is the regulation of membrane potential by allowing ion permeation. In class-switched memory B cells (CD27^+^IgD^−^), the amplitude of the K_V_1.3 current was found to be significantly greater compared to naive (CD27^−^IgD^+^) and CD27^+^IgD^+^ memory B cells, and its specific blockade only inhibited the proliferation of the class-switched group [33]. K_Ca_3.1 is a calcium-gated K^+^ channel, which is activated following B cell receptor (BCR) stimulation, and, together with K_V_1.3, it maintains the membrane potential required for sustained calcium entry during lymphocyte activation [34,35,36]. Specific blockade of K_Ca_3.1 decreased the proliferation rate of the naive and IgD^+^CD27^+^ memory B cells, in which K_Ca_3.1 is dominant over K_V_1.3 in membrane potential control [33].

Several reports exist on the expression and function of L-type calcium channels (Ca_V_1.1–1.4) in lymphocytes following T and B cell receptor stimulation, which are activated by an unknown, membrane-potential-independent mechanism [37,38,39,40,41], however, direct electrophysiological recordings are lacking. The store-operated calcium channel, ORAI1, is activated by TCR/BCR stimulation and Ca^2+^ store depletion and has a role in lymphocyte proliferation and cytokine production as well [42,43,44,45,46,47]. 

Sodium-permeable channels are not well-described in lymphocytes, although there are a few studies suggesting that sodium influx may play a role in B and T cell responses, as it is thought that sodium influx could decrease the chance of calcium overload, preventing cell death [48]. These channels include amilorid-sensitive sodium channels (ENaC), TRP channels and purinergic receptors [34,48,49,50]. 

Based on flow cytometry and patch-clamp measurements, the membrane potential of human T and B lymphocytes was sensitive to both the removal of extracellular Na^+^ and TTX application, implying the presence of voltage-gated sodium (Na_V_) channels, but the isoform involved was not identified [51,52,53]. Jurkat T cells were found to express Na_V_1.5 at appreciable levels with Na_V_1.9 expressed at much lower levels [54], while another study also found a functional role of Na_V_1.5 channel in CD4^+^ T cells [20]. A TTX-resistant Na_V_ channel was also found in T cells stimulated with an antigenic peptide, but the isoform was not identified [55].

Thus, several lines of evidence indicate the presence of Na_V_ channels in lymphocytes; however, until now there has been no comprehensive electrophysiological study performed focusing on these channels in B cells. In the current study, we have found and characterized a voltage-gated sodium current in human B lymphocytes by electrophysiological and molecular biology methods. In addition, we explored the potential canonical function of the Na_V_ channels creating the current, that is, whether they contribute to the control and maintenance of the membrane potential.

## 2. Materials and Methods

### 2.1. B Cell Isolation

A total of 25−30 mL of sodium heparin anticoagulated peripheral whole blood was taken from 8 healthy volunteers on the day of the patch-clamp measurements. These individuals were staff members from the Departments of Biophysics and Cell Biology and Laboratory Medicine who underwent a detailed medical history, physical examination and routine laboratory tests and were free of acute cardiovascular, metabolic, inflammatory diseases or cancer. First, the mononuclear layer was separated, using Histopaque-1077 (Sigma-Aldrich, St. Louis, MO, USA). Next, B cells were isolated using human Pan B cell Isolation Kit employing magnetic beads (Miltenyi Biotec, Bergisch Gladbach, Germany). The excess of B cells (which were not used up for electrophysiological measurements) were placed in TRI Reagent (Molecular Research Center, Cincinnati, OH, USA) and saved for qPCR testing.

### 2.2. Electrophysiology

Whole-cell currents of voltage-clamped cells were recorded by manual patch-clamp electrophysiology according to standard protocols using Axopatch 200B amplifiers connected to a computer via Digidata 1550B digitizers (Molecular Devices, San Jose, CA, USA). Pipettes were pulled from GC 150F-15 borosilicate glass capillaries (Harvard Apparatus, Holliston, MA, USA) in four stages with tip diameters between 0.5 and 1 μm with 3–5 MΩ resistance. Immediately before the measurement, the cells were maintained in the recording petri dish in bath solution (i.e., control solution) consisting of 145 mM NaCl, 5 mM KCl, 1 mM MgCl_2_, 2.5 mM CaCl_2_, 5.5 mM glucose and 10 mM HEPES, pH = 7.35 (titrated with NaOH). For the recordings, the composition of the solution used in patch pipette (internal solution) was 145 mM KF, 5 mM sodium L-aspartate, 11 mM EGTA, 10 mM HEPES, 2 mM MgCl_2_ and 1 mM CaCl_2_, pH = 7.22, titrated with KOH. For the testing of voltage-gated sodium currents, sodium-free solution (145 mM choline chloride, 5 mM KCl, 1 mM MgCl_2_, 2.5 mM CaCl_2_, 5.5 mM glucose and 10 mM HEPES, pH = 7.35, titrated with choline base) and TTX (5, 15, 50 and 150 nM in control solution) were used. For the membrane potential measurements, a high concentration potassium solution (150 mM KCl, 10 mM HEPES, 5.5 mM glucose, 1 mM CaCl_2_ and 1 mM MgCl_2_, pH = 7.35, titrated with KOH) was used in addition to the control and sodium-free solutions. Solution exchange was achieved by using a gravity-flow system with continuous excess fluid removal. To avoid the changing of junction potentials during solution changes, the reference electrode, placed in a dish containing internal solution, was connected to the bath solution with an agar bridge. For the voltage-clamp measurements, a holding potential of −100 mV, for current-clamp recordings 0 nA current injection, were used. Patch-clamp data were acquired with pClamp10 (Molecular Devices, San Jose, CA, USA). In general, currents were low-pass-filtered using the built-in analog four-pole Bessel filters of the amplifiers and sampled at 20 kHz. Before analysis, whole-cell current and membrane potential traces were digitally filtered (five-point boxcar smoothing). Experiments were done at room temperature ranging between 20 and 24 °C. Clampfit 10.7 (Molecular Devices, CA, USA) and Graphpad Prism 7 (Graphpad, San Diego, CA, USA) were used for data display and analysis. The G-V and SSI curves were fit with the Boltzmann equation $\left(Y=1/\left(1+\exp\left(\left(V_{1/2}-X\right)/k\right)\right)\right)$, wherein *k* is the slope factor and *X* is the set membrane and holding potential respectively. To determine the rate of channel recovery from inactivation, we fit the curve with a single rising exponential equation (Y=(1 − C)×(1−exp(−t/τ))+C) and obtained the time constant *τ*.

### 2.3. RT-qPCR

Total RNA, including messenger RNAs (mRNA), was isolated from B-cells using TRI Reagent according to the manufacturer’s recommendations. The concentration and purity of separated RNA samples were verified by a NanoDrop 2000 spectrophotometer (Thermo Scientific, Wilmington, DE, USA), and samples were stored at −80 °C before real-time quantitative polymerase chain reaction (RT-qPCR) analysis. For the quantification of the expression of selected voltage-gated sodium channel alpha subunit mRNAs (SCN1A-SCN11A), complementary DNA (cDNA) synthesis was performed using a High-Capacity cDNA Reverse Transcription Kit (Applied Biosystems, Vilnius, Lithuania) according to the manufacturer’s recommendations. RT-qPCR was performed using a LightCycler 480 SYBR Green I Master mix (Roche Diagnostics, Mannheim, Germany) with gene-specific primers (10 µM, Integrated DNA Technologies, Leuven, Belgium). The reactions were run on a LightCycler 96 RT-qPCR instrument (Roche Diagnostics). The reactions were incubated at 95 °C for 10 min, followed by 40 cycles of 95 °C for 10 s and 60 °C for 1 min [56]. The reference gene RPLP0 (36B4) was used for normalization. All measurements were run in duplicate. Cycle threshold (Ct) values were automatically determined by the instrument. Ct values over 35 were graded as noise. Sequences of these oligonucleotides are listed in Supplementary Table S1.

## 3. Results

### 3.1. Voltage-Gated Sodium Currents Can Be Detected in Human B Cells

**Figure 1:**
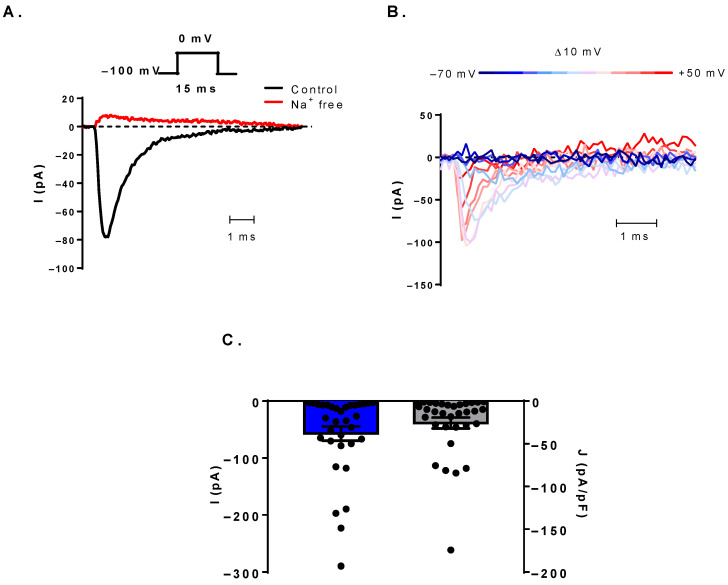
Voltage-gated sodium currents are present in human B lymphocytes. (**A**) An inward current evoked by a depolarizing step from −100 mV to 0 mV. Black line is for the current recorded in control solution, while red trace represents the current recorded in Na^+^-free solution. (**B**) The voltage-dependence of the current is shown from −70 to +50 mV in + 10 mV increments. The negative clamped potentials are in the shades of blue, and the positive ones are in the shades of red. (**C**) Summary of the peak current (blue bar, left Y-axis) and current density (gray bar, right Y-axis) values obtained at 0 mV depolarizations. Bar heights indicate mean ± SEM.

As depicted in Figure 1A, a significant fraction of patch-clamped human B cells displayed inward currents with fast activation and inactivation kinetics using 15 ms-long depolarizing steps to 0 mV from a holding potential of −100 mV every 15 s in whole-cell configuration. The current was abolished while the cells were perfused with Na^+^-free external solution, indicating that Na^+^ ions are the charge carriers. The current kinetics implied the presence of Na_V_ channels, which was tested by checking the voltage-dependence of the current kinetics with an I-V protocol. Depolarizing pulses ranging from −70 to +50 mV in 10 mV steps evoked currents with increasingly fast activation and inactivation kinetics, which is characteristic of Na_V_ channels (Figure 1B). We could detect Na_V_ current in 33 out of 85 measured cells. In several cells, the detection of the small current amplitude required the use of an averaging protocol, which yielded the average of 100 sweeps acquired at 4.8 Hz to improve the signal-to-noise ratio. The identity of the current was always verified by switching back and forth between Na^+^-free and control external solutions. The current amplitude and density varied among B cells with a mean of −57.0 ± 12.6 pA and −25.7 ± 6.4 pA/pF, respectively (N_donor_ = 8, n_cell_ = 33) (Figure 1C).

### 3.2. Biophysical Characterization of the Na_V_ Channels

**Figure 2:**
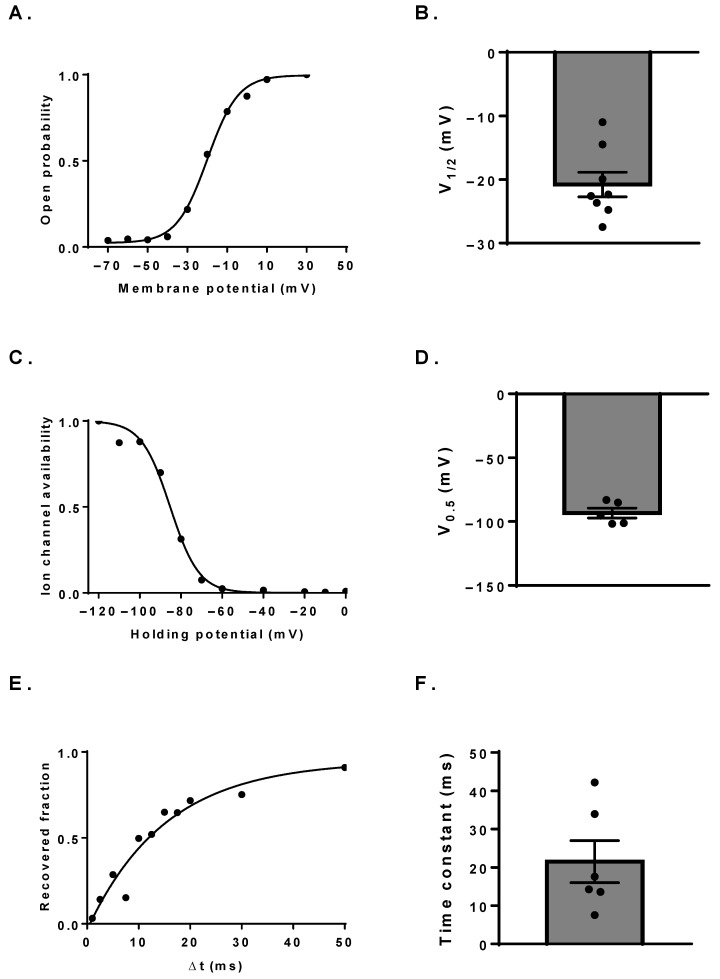
Biophysical characteristics of Na_V_ channels expressed in B cells. (**A**) A representative G-V curve from an individual cell. (**B**) Half-maximal activation potentials of the individual cells determined from G-V curves. (**C**) Voltage-dependence of the steady-state inactivation of a representative cell determined by depolarizing pulses to 0 mV from the indicated holding potentials. (**D**) Half-inactivation potentials determined from SSI curves. (**E**) Kinetics of the recovery from inactivation of a representative cell determined by depolarizing pulse pairs to 0 mV separated by various recovery time intervals (Δt) at −100 mV. (**F**) Summary of the recovery time constants. Throughout the figures, bar heights indicate mean ± SEM and the black symbols represent data obtained from individual cells.

We have further investigated the biophysical characteristics of the Na_V_ channels in the human B cell membrane in an attempt to identify their subtype. Using appropriate voltage protocols, we determined the voltage-dependence of steady-state activation and inactivation and the kinetics of recovery from inactivation as well. Boltzmann fits to the conductance-membrane potential relationship curves (G-V) yielded a half-maximal activation potential (V_1/2_) of −20.8 ± 1.9 mV (n = 8, Figure 2A,B). The voltage-dependence of ion channel availability was determined from the steady-state inactivation curve (SSI). Cells were held at holding potentials ranging from −120 to +20 mV in 10 mV increments for 5 s, then the fraction of available channels was measured by a depolarizing pulse to 0 mV. Based on Boltzmann fits to the SSI curves, the resting membrane potential, at which half of the channels are available for activation (i.e., half-inactivation potential, V_0.5_), was −93.4 ± 3.9 mV (n = 5, Figure 2C,D). The rate of recovery from inactivation was measured by depolarizing pulse pairs to 0 mV separated by increasing time intervals (Δt) ranging from 1 ms to 300 ms at −100 mV. The recovered channel fraction was determined as the ratio of the current amplitudes in the second pulse to that in the first pulse. The recovery time constant was obtained by a single-exponential fit to the measured points. The mean time constant was τ = 21.5 ± 5.5 ms (n = 6, Figure 2E,F), but the data suggest the presence of multiple channel types, as time constants varied over a wide range of values among the cells (7.5−42 ms).

### 3.3. The Sodium Current Is TTX-Sensitive

**Figure 3:**
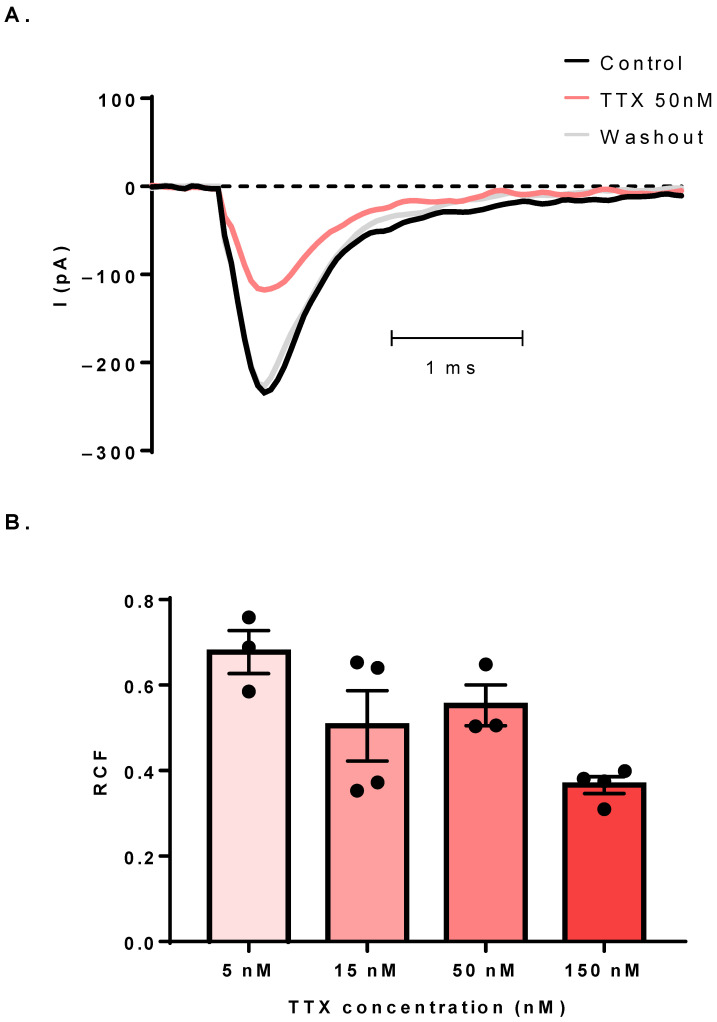
The sodium current is TTX-sensitive. (**A**) The effect of 50 nM TTX on the Na_V_ current. The control trace (black) was recorded in normal extracellular solution, and TTX (red, equilibrium block) caused an inhibition of the inward current, which was reversed by returning to the control solution (gray, washout). All traces were recorded at 0 mV. (**B**) The concentration-dependence of the remaining current fractions (RCF) was at 0 mV. Black symbols indicate the RCFs of individual cells, which were calculated by the equation RCF = I_TTX_/I_control_, where I_TTX_ is the current amplitude measured with TTX perfusion, and I_control_ is the current amplitude measured in control solution. Bar heights indicate mean ± SEM.

We have performed pharmacological experiments to gain more information about the identity of the expressed Na_V_ channels by applying TTX perfusion in the extracellular solution at 5, 15, 50 and 150 nM concentrations. Figure 3A shows representative Na_V_ current traces in control solution, in the presence of 50 nM TTX and a subsequent washout. The application of TTX caused a reversible inhibition of the current, which implied the presence of the TTX-sensitive Na_V_ isoforms. We then obtained the dose-response of TTX with the above-mentioned concentrations (5–150 nM) in cells with sufficiently large currents. The remaining current fractions (RCF) at 0 mV were 0.67 ± 0.05 (n = 3), 0.50 ± 0.08 (n = 4), 0.55 ± 0.05 (n = 3) and 0.36 ± 0.02 (n = 4), respectively (Figure 3B). The extent of blockage at high TTX concentrations was less than expected, which suggested the contribution of TTX-resistant Na_V_ isoforms to the current. 

### 3.4. Membrane-Potential Measurements Show a Potential Canonical Function of Na_V_ Channels

**Figure 4:**
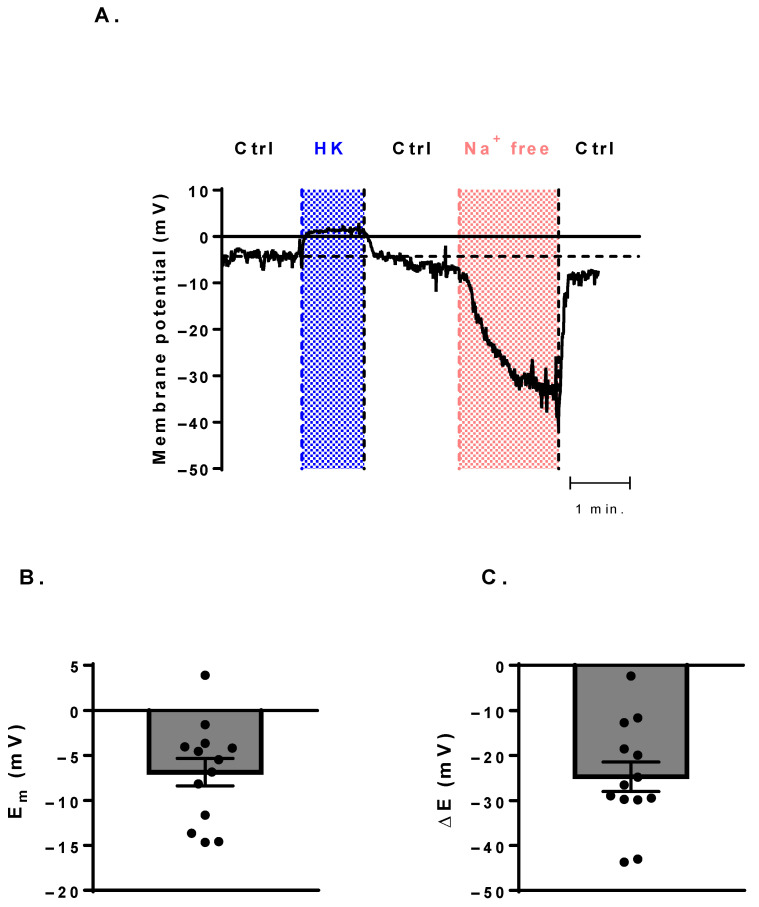
Membrane-potential measurements of the B cells. (**A**) The effect of the HK (blue area) and Na^+^-free (choline) solutions (red area) on the resting membrane potential. The horizontal dashed line indicates the resting membrane potential at the beginning of the recording, measured in control solution. (**B**) Distribution of the resting membrane potentials measured in control conditions. (**C**) Effect of Na^+^-free solution on the membrane potential (ΔE = E_Na+ free_ − E_m_). Bar heights indicate mean ± SEM, and the black symbols represent data obtained from individual cells.

In order to assess if the expressed Na_V_ channels have a canonical function, that is, whether they take part in the maintenance of the resting membrane potential, we have measured the resting membrane potential with I = 0 current-clamp mode (Figure 4). The average resting membrane potential (E_m_), measured with control solution was −6.8 ± 1.5 mV (n = 13, Figure 4B). The perfusion of high-concentration potassium solution (HK) was used as a positive control, and it indeed caused a reversible shift of the membrane potential close to 0 mV in each cell. The application of the Na^+^-free choline chloride-based solution made the membrane potential more negative (ΔE = −24.7 ± 3.2 mV, n = 13) reversibly, indicating the contribution of the Na_V_ channels to the control of the resting membrane potential of B cells (Figure 4A,C).

### 3.5. RT-qPCR Confirms Multiple Na_V_ Channel Types in B Cells

**Figure 5:**
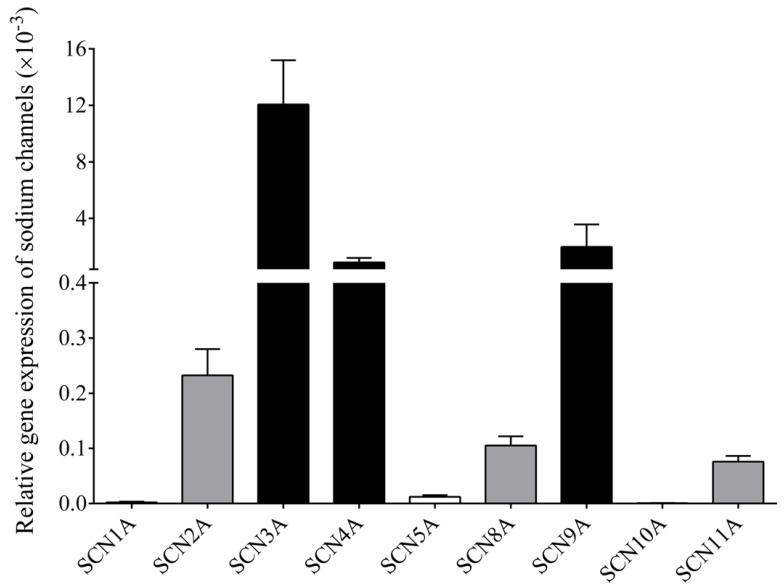
Analysis of the expression of multiple Na_V_ channels by RT-qPCR in B cells.The relative expression of nine different Na_V_ channels in human B cells was calculated based on the average copy number of the target genes normalized to the reference gene RLPLP0 (36B4). SCN3A, SCN4A and SCN9A (Na_V_1.3, 1.4 and 1.7) subtypes were found to be highly expressed (black bars), while only low expression of SCN2A (Na_V_1.2), SCN8A (Na_V_1.6) and SCN11A (Na_V_1.9) (gray bars) was detected. In addition, no expression was observed in SCN1A (Na_V_1.1), SCN5A (Na_V_1.5) and SCN10A (Na_V_1.8) channels (white bars). Mean ± SEM are depicted, n = 6/group.

We analyzed the expression of nine different Na_V_ channels in isolated B cell samples of six healthy individuals using RT-qPCR (Supplementary Figure S1). Of these, six channels, Na_V_1.2, 1.3, 1.4, 1.6, 1.7 and 1.9 (the corresponding genes being SCN2A, SCN3A, SCN4A, SCN8A, SCN9A and SCN11A, respectively) showed a detectable amount of specific mRNA in comparison to RPLP0 reference gene. The SCN3A, SCN4A and SCNA9 subtypes showed the highest expression, while SCN2A, SCN8A and SCN11A were expressed at lower levels, and SCN1A (Na_V_1.1), SCN5A (Na_V_1.5) and SCN10A (Na_V_1.8) channels could not be detected as they were showing Ct values over 35 (Figure 5).

## 4. Discussion

Although several previous studies implied the existence of Na_V_ channels in B lymphocytes, direct electrophysiological evidence has been lacking so far. In our study, we demonstrate the expression of voltage-gated Na^+^ channels in human B cells using electrophysiological and molecular biology methods. A significant fraction (~40%) of patch-clamped B cells exhibited voltage-gated Na^+^ currents. (Figure 2C). In contrast, a TTX-sensitive sodium current was detectable only in about 3% of human T cells, although no special effort was devoted to detecting them in that study, so the actual fraction may be higher [19]. By RT-qPCR we could detect TTX-sensitive (Na_V_1.2, 1.3, 1.4, 1.6 and 1.7) and TTX-insensitive (Na_V_1.9) Na_V_ transcripts with Na_V_1.3, 1.4 and 1.7 showing dominant expression, while Na_V_1.9 was also detectable but with a lower expression. This can account well for the pharmacological results, as the highest applied concentration of TTX (150 nM) did not block the sodium current to the extent that would have been expected in the presence of only TTX-sensitive Na_V_ channels in the membrane [57,58]. The estimated IC_50_ for TTX inhibition calculated from the RCF at 150 nM TTX yields 84 nM, which is many times higher than the IC_50_ values reported for TTX-sensitive channels. This supports the hypothesis of TTX-resistant channels also being present in the B cell membrane, but in lower numbers and showing cell-to-cell variability. Furthermore, the expression pattern of ion channels is known to change in various differentiation states of the cells. Such a change in the relative expression of the K^+^ channels in human T and B cells is well-documented [33,59]. A similar change was reported for the expression of Na_V_ channels in dendritic cells: during maturation, the downregulated expression of Na_V_ channels was accompanied by an upregulation of K_V_ channels [25]. A follow-up study by the same group demonstrated that the high expression of Na_V_ channels was restricted to an even smaller subset of immature dendritic cells (CD1a+), implying a strong link to a differentiation state [14]. A similar shift in voltage-gated cation channel expression was also reported for differentiating chondrocytes [24]. In addition, external factors, such as injury, could also initiate alterations in the expression pattern, as was shown for astrocytes, in which mechanically induced gliosis led to a switch from TTX-sensitive to TTX-resistant Na^+^ current [60]. As in our study we did not separate B cells by differentiation markers (e.g., CD27), the investigated population was heterogeneous in this respect, and this may have contributed to the observed high variability in TTX-sensitivity. This may also explain why we could detect Na_V_ expression only in a certain fraction of B cells: measurable Na_V_ currents may be limited to specific B cell subsets or differentiation states, which represent a smaller part of the population. This should be investigated in the future to further elucidate the functional role of Na_V_ channels in B cells.

Based on the examined biophysical parameters, we cannot unambiguously differentiate among the Na_V_ channels. Most gating parameters show a dependence on the expression system, since factors, such as membrane lipid composition and associated auxiliary subunits, can significantly modulate them. Consequently, most parameter ranges reported in the literature overlap for the different Na_V_ subtypes, preventing precise identification simply based on these values. For example, the average half-maximal activation potential we obtained in B cells (−20.8 ± 1.9 mV) is quite similar for most Na_V_ channels, with only Na_V_1.9 activating at more negative potentials (V_1/2_ around −40 mV) [61,62,63,64]. The heterogeneity in the measured recovery time constants (range: 7.5—42 ms) is also in accordance with the RT-qPCR results, indicating the presence of multiple subtypes, as this parameter shows great variability among Na_V_ channels (e.g., Na_V_1.3 and 1.4, τ = 8−20 ms [65]; Na_V_1.7, τ ≈ 150 ms [66]). 

We have examined the potential canonical function of the detected Na_V_ channels and concluded that they contribute to the regulation of the resting membrane potential, as the application of the sodium-free solution caused a reversible hyperpolarization from a rather depolarized resting membrane potential in each cell. Reported lymphocyte membrane potential values span a very wide range from quite negative (−70 to −60 mV [67,68]) to intermediate (−44 mV [34]) to much more positive (−7 to −12 mV [69,70]). This large variability is likely to stem from the different measuring methods used and the varying cell populations examined. The possibly more positive membrane potential of B cells is masked by their low abundance in mixed lymphocyte populations if T cells have more negative membrane potentials. Although at this membrane potential most of the Na_V_ channels are in a steady-state inactivated state (at 0 mV the Na_V_ channel availability was 0.05 ± 0.02), the high impedance of the cell membrane combined with the small cytoplasmic volume of B cells may result in significant membrane potential changes due to even small currents. In principle, the removal of the external Na^+^ may induce membrane potential changes indirectly by interfering with the Na^+^-dependent electrogenic transport mechanisms, but this possibility was ruled out, and the direct effect of Na_V_ channels was demonstrated at similarly depolarized membrane potentials in dendritic cells, supporting the validity of our hypothesis [14]. 

The physiological function of Na_V_ channels in lymphocytes is still not clear. Some studies suggested their role in membrane potential control in human T and B cells, but in T cells, it was shown by TTX application that they are not required for mitogenesis [19,51,52,53]. In Jurkat T cells, the presence of Na_V_ channels was associated with migration, as it was significantly inhibited by the application of TTX in a Matrigel invasion assay [54]. Na_V_1.5 was shown to be essential for the positive selection of CD4^+^ T cells, as its pharmacological inhibition or knockdown inhibited the selection process [20]. The Na_V_ isoforms that we found by PCR to show the highest expression are not mentioned in any publications pertaining to lymphocyte ion channels.

Immune cells show similarities in their channelomes and the changes in expression levels during differentiation. For example, the voltage-gated K_V_1.3 channel is expressed in quiescent macrophages, microglia, dendritic cells and T and B lymphocytes and is significantly upregulated in certain subtypes of these cells following activation [33,59,71,72]. Potassium channels are known to be essential for activation-associated sustained Ca^2+^ influx by maintaining a hyperpolarized membrane potential. We propose that, similarly to the highly inflammatory CD1a+ dendritic cell subtype, Na_V_ channels in quiescent lymphocytes, and B cells in particular, may help in maintaining a depolarized membrane potential preventing unnecessary activation. The low steady-state activity of Na_V_ channels accompanied by low expression of K_V_ and K_Ca_ channels may stabilize this depolarized state. Then, following a sufficiently strong activating stimulus, a switch in channel expression occurs with the downregulation of Na_V_ and upregulation of K_V_ channels, which then establishes the conditions for calcium signaling.

In light of the small sodium current amplitudes, the possible non-canonical functions of Na_V_ channels in B cells can also be considered, which are not related to ion permeation and membrane potential control. Such functions were described for K_V_2.1 and 2.2 channels in neurons, which form large non-conducting clusters to create junctions between the plasma membrane and the endoplasmic reticulum serving as trafficking hubs [73,74]. Similarly, an ion permeation-independent functional role of K_V_1.3 channels was demonstrated in the proliferation of vascular smooth muscle cells, in which the voltage-gating mechanism of the channels was sufficient to maintain the proliferative pathway without K^+^ fluxes [75]. Although such functions cannot be ruled out, based on the strong effect of Na^+^ removal on the membrane potential and the documented expression changes in other cell types upon differentiation, we find a similar canonical function in B cells more likely.

In summary, our study proclaims that the presence of Na_V_ channels in human B cells needs to be considered in future pharmacological and functional studies, as they are present in a significant fraction of cells. Exploring Na_V_ channel expression in B cell subsets of various differentiation states may provide tools to selectively target immune functions by pharmacological means.

## Data Availability

The datasets of the current study are available from the corresponding author on reasonable request.

## Supplementary Materials

The following supporting information can be downloaded at: https://www.mdpi.com/article/10.3390/cells11071225/s1, Figure S1: Representative RT-qPCR curves of 9 Na_V_ specific mRNAs in human B cells; Table S1: Oligonucleotide sequences, used for RT-qPCR

## Abbreviations

ATP: adenosine triphosphate, BCR: B cell receptor, cDNA: complementary DNA, Ct: cycle threshold, G-V: conductance-membrane potential relationship, mRNA: messenger RNA, NaV: voltage-gated sodium channel, RCF: remaining current fraction, RT-qPCR: real-time quantitative polymerase chain reaction, SOCE: store-operated calcium entry, SSI: steady-state inactivation, tau: recovery time constant, TCR: T cell receptor, TTX: tetrodotoxin, V_0.5_: half-inactivation potential, V_1/2_: half-maximal activation potential.
