# Supplementary material for: Functional Voltage-Gated Sodium Channels Are Present in the Human B Cell Membrane
Source: Cells. 2022 Apr 5;11(7):1225. doi: 10.3390/cells11071225 (PMC8998058; doi:10.3390/cells11071225)
Supplement: Supplementary file 1 [file cells-11-01225-s001.zip › cells-1646044-supplementary.pdf]

cells MDPI

Supplementary material

Functional voltage-gated sodium channels are present in the human B cell membrane

Adam Feher[1], Marianna Pócsi[2], Ferenc Papp[1], Tibor G. Szanto[1], Agota Csoti[1], Zsolt Fejes[2], Béla Nagy Jr.[2], Balázs Nemes[3], and Zoltan Varga[1*]

[1] Department of Biophysics and Cell Biology, Faculty of Medicine, University Debrecen, Debrecen, Hungary; veze@med.unideb.hu
[2] Department of Laboratory Medicine, Faculty of Medicine, University of Debrecen, Debrecen, Hungary; nagy.bela@med.unideb.hu
[3] Department of Organ Transplantation, Institute of Surgery, Faculty of Medicine, University of Debrecen, Debrecen, Hungary; nemes.balazs@med.unideb.hu

* Correspondence: veze@med.unideb.hu

Table S1. Oligonucleotide sequences, used for RT-qPCR.

Messenger RNAs	Forward primers for RT-qPCR (5' - 3')	Reverse primers for RT-qPCR (5' - 3')	Amplicon length
SCN1A	AGTAGTGAATCGGATCTGGAAG	CCACTACGGGCTGTTCTTCT	112 nt
SCN2A	CACCCCTGCCCTTTACATTT	AGTTGGTAAGAATCGTGCACA	113 nt
SCN3A	GCAACAGGAAGAAGCTCAGG	AACAGCTCTCCTAACCCACC	84 nt
SCN4A	AAGCCTCAGAAGCCAATTCC	CACGAGGTCATACACCATGC	63 nt
SCN5A	CACAGCTGGTTCGAGACATT	CGGCATACTCAAGCAGAACC	118 nt
SCN8A	AACAGCACGGTGGACTGC	AATTTCCACCTCAGTTGTAGCC	102 nt
SCN9A	CAACTTTTAAGGGATGGACGA	TCATATTTGGGCTGCTTGTCT	76 nt
SCN10A	ATCGTCACTGTGAGTCTGCT	CAGGATTTGGCCAGCTTGAA	104 nt
SCN11A	GGGTAGCCTTCGGATTTGGA	AATGAGGGTGGTCACAGAGA	89 nt
RPLP0 (36B4)	ATGCAGCAGATCCGCATGT	TCATGGTGTTCTTGCCCATCA	64 nt

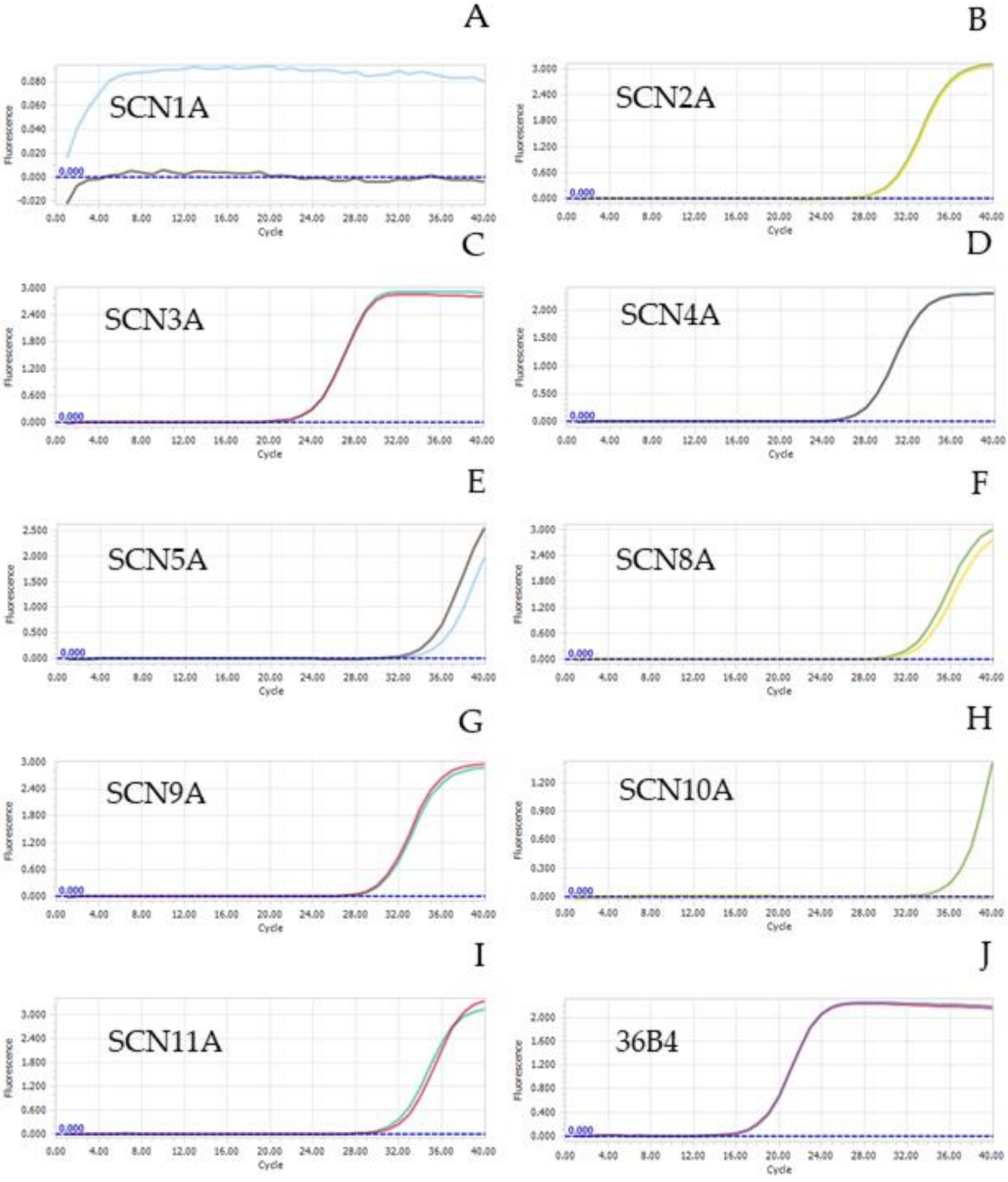

Figure S1. Representative RT-qPCR curves of 9 Nav specific mRNAs in human B cells.

Representative RT-qPCR curves of 9 Nav specific mRNAs in human B cells (A-I). Based on the cycle threshold (Ct) values during quantification, we determined which sodium channels could be expressed by healthy B cells showing a detectable level of

mRNA. Ct values over 35 were graded as noise. The following typical mean Ct values of mRNA expression for each sodium channel in brackets were observed in one of the enrolled subjects: 40.0 (SCN1A), 29.79 (SCN2A), 23.38 (SCN3A), 27.77 (SCN4A), 35.93 (SCN5A), 32.26 (SCN8A), 29.89 (SCN9A), 40.0 (SCN10A), and 31.53 (SCN11A). The reference gene 36B4 (RPLP0) with a mean Ct value of 18.0 was used for normalization (J).
